# Evaluating Severe Therapy-Resistant Asthma in Children: Diagnostic and Therapeutic Strategies

**DOI:** 10.3390/medicina60111799

**Published:** 2024-11-02

**Authors:** Andrew Bush

**Affiliations:** Department of Paediatric Respiratory Medicine, Royal Brompton Hospital, Sydney Street, London SW3 6NP, UK; a.bush@imperial.ac.uk; Tel.: +44-207-352-8232

**Keywords:** adherence, allergies, asthma attacks, EILO, inhaled corticosteroid, long-acting beta-2 agonist, monoclonal antibodies, obesity, prednisolone, SMART regime

## Abstract

*Introduction*: Worldwide, asthma is the most common non-communicable respiratory disease and causes considerable morbidity and mortality. Most people with asthma can be treated effectively with low-dose medications if these are taken correctly and regularly. Around 10% of people with asthma have an uncontrolled form of the disease or can only achieve control with high-dose medications, incurring disproportionately high health care costs. *Areas Covered*: PubMed and personal archives were searched for relevant articles on the definition, management and pharmacotherapy of severe asthma. The WHO classification of severe asthma and the treatment levels encompassed in the definition are discussed. Most children and young people referred for consideration of ‘beyond-guidelines therapy’ can in fact be managed on standard treatment after a multi-disciplinary team assessment focusing on ensuring correct basic management, and these steps are described in detail. Options for those with true therapy-resistant asthma are described. These include monoclonal antibodies, most of which target type 2 inflammation. *Expert Opinion*: Getting the basics right is still the most important aspect of asthma care. For those with severe, therapy-resistant asthma, an increasing number of life-transforming monoclonals have been developed, but there is still little understanding of, and a paucity of treatment options for, non-eosinophilic asthma.

## 1. Introduction

Worldwide, asthma is one of the most important non-communicable respiratory diagnoses in children. The World Health Organization (WHO) estimate that asthma affects more than 262 million people worldwide and causes nearly half a million deaths a year [1], many, tragically, in children. Although cheap, low-dose medications are dramatically effective in most children and young people (CYP), around 5–10% have severe asthma [2,3], defined by the WHO as ‘uncontrolled asthma which can result in risk of frequent severe exacerbations (or death) and/or adverse reactions to medications and/or chronic morbidity’. The WHO acknowledges three categories of severe asthma [4]:

- Untreated severe asthma due to misdiagnosis, lack of access to medical care or non-compliance with treatment, usually in a low- and middle-income settings. It should be noted that there is an underclass of poverty where medications may not be available, even in the most affluent countries. In this group, the main problem is usually that patients cannot access controller treatments. Shamefully, worldwide, this is the largest group of severe asthma patients. Political action is the answer for this group.
- Difficult-to-treat severe asthma resulting from comorbid conditions or adverse environmental circumstances.
- Treatment-resistant severe asthma (STRA), which includes patients in whom control is not achieved despite high-dose therapy or for whom control can only be maintained with such therapy.

The solution to untreated severe asthma is political, ensuring that every CYP has access to the WHO-approved list of asthma medications [5,6] and an appropriate spacer, which can be made from a plastic bottle [7]. The effects of ensuring widespread access to low-dose inhaled corticosteroids (ICSs) are dramatic [8]. This group of patients will not be discussed further; the focus of this manuscript is difficult-to-treat asthma, and the much rarer STRA, in a high-income setting.

Properly, the title of this article should include the words ‘the child with asthma-like symptoms not responding to treatment for asthma’. The scope of the article is school-age children; preschool wheeze is comprehensively covered elsewhere [9]. Before even considering additional therapies, it is essential to review the basics; two important questions should be asked first:

- What is the positive evidence that this child has asthma at all?
- Is there evidence that there is a different or additional diagnosis?

This invited review will set out the stepwise process by which these children should be assessed and how to determine what is the right treatment option. The special circumstances of obesity asthma and the asthma attack-prone child are also considered in specific sections. The review is timely in that we are now accumulating more data on biologics in paediatric asthma.

### 1.1. Does the Child Have Asthma at All?

An asthma diagnosis is all too often unconfirmed by objective tests and thus incorrect [10,11]. There is no one definitive diagnostic test for asthma, but a safe principle is that the more negative tests that are performed, the less likely the diagnosis is to be correct. The positive evidence for an asthma diagnosis should always be documented, no matter how eminent the diagnostician.

### 1.2. Case Study

An 11-year-old boy was referred as STRA for a biological. He had daily respiratory symptoms which were poorly responsive to treatment but was never admitted to the emergency department. Examination revealed biphasic stridor and no wheeze. Investigations showed a normal FeNO and blood eosinophil count and no evidence of allergic sensitization. A flow volume curve showed attenuated peak inspiratory and expiratory flow rates suggestive of large airway obstruction. Contrast CT scanning established the diagnosis of double aortic arch, which was treated surgically with relief of symptoms.

Asthma is a clinical diagnosis comprising wheeze, breathlessness, chest tightness and sometimes cough [12]. The cardinal symptom, wheeze, is (a) frequently confused with other respiratory noises by parents and child [13,14,15,16], and (b) non-specific. Any cause of intra-thoracic airway narrowing can cause wheeze. These include intraluminal secretions, tumours or foreign bodies; airway wall diseases, such as tracheobronchomalacia or complete cartilage rings; and external compression, for example, by tuberculous lymph nodes or a vascular ring. A key question is, has the presence of wheeze been documented by an experienced health care professional? Note that in one study, emergency care nurses only poorly identified wheeze [15]. Physical examination is usually normal during an elective review when the child is well, but may give a clue that an underlying, non-asthma diagnosis should be sought.

The key objective tests that need to be considered are for atopy, variable airflow obstruction and airway inflammation; none are diagnostic of asthma. Although in many parts of the world, non-atopic, non-eosinophilic asthma is more common than eosinophilic [17], in high-income settings, non-responsive asthma is unusual in the non-atopic. A history of atopic dermatitis, allergic rhinitis and food allergy should be sought, and either, or preferably both, skin prick and specific immunoglobulin sIgE tests for aeroallergens and foods should be performed (sIgE and SPTs overlap but do not provide identical information [18]). Airflow obstruction varying over time and with treatment should also be sought. If the child has airflow obstruction seen on either peak flow or, better, spirometry, the acute response to a short-acting β-2 agonist (SABA) is documented. However, most children with asthma have normal lung function between attacks, so this is not useful. Home peak flow or spirometry monitoring is another test, preferably with objective recording of the measurements, since data fabrication is common after more than two weeks of monitoring [19]. The family should be provided with a SABA in a suitable device and try to measure lung function; if they believe a SABA is needed, before and after administration; and before and after exercise. Airway challenge tests are not routine, but a field exercise test may be helpful, albeit non-specific [20]. Histamine or methacholine challenge, if negative in a symptomatic child, effectively excludes asthma as the cause of the symptoms; a positive challenge is less useful, since normal children may be ‘positive’. Indirect evidence of eosinophilic airway inflammation should be sought by measuring peripheral blood eosinophil count and exhaled nitric oxide (FeNO). Both may be elevated in atopic, non-asthmatic children, and blood eosinophils may be elevated with parasitic infestations. More direct evidence is induced or spontaneously expectorated sputum eosinophilia, but in most centres, this is a research technique. Table 1 summarizes objective testing for asthma.

### 1.3. Is There Evidence of an Alternative or Accessory Diagnosis?

The differential diagnosis of asthma is wide; testing for alternative diagnoses should be focused; not all tests can or should be performed on all children. Table 2 summarises the diagnostic clues that further investigation is needed based on history, examination and the chest radiograph.

## 2. Approach to the Child with Apparent Severe, Therapy-Resistant Asthma (STRA)

Definitions of STRA have been proposed based largely on level of medication [21,22] (Table 3). A seminal study casts doubt on the arbitrary levels of inhaled corticosteroids (ICSs) which define severe asthma. The BADGER study [23] addressed the question, in children who were symptomatic on 100 mcg twice daily of Fluticasone, of whether the best strategy was to add a long-acting β-2 agonist (LABA) or a leukotriene receptor antagonist (LTRA) or to increase the ICS dose to 250 mcg twice daily. Most responded best to adding a LABA, and very few showed any improvement with the bigger ICS dose. Thus, the escalation of ICS dose to levels at the higher steps of the guidelines is for the most part not justified. Another study posed the question of whether, in a child symptomatic on ICSs and LABAs, it is best to add azithromycin or an LTRA [24]. The study ended in futility because most of those screened either did not have asthma or were not taking treatment. Finally, in VOYAGE, a randomized, placebo-controlled study of Dupilumab [25] (below), the effect of being in a clinical trial and being on placebo was a reduction in the annualized asthma attack rate from 2.16 to 0.67, a fall to just above 30% of the rate in the run-in year with no change in pharmacotherapy [26]. Therefore, it is unsurprising that our experience is that most children referred to our quaternary service for ‘beyond guideline’ therapy in fact just needed to get the basics right [27,28,29,30].

### 2.1. The First Step: A Multidisciplinary Team Assessment

Assuming the diagnosis of asthma is secure, the next question is, what is it about this child and his/her asthma which means there is not the anticipated response to treatment? A detailed assessment places the child into one of three categories, which may overlap:

- Difficult asthma, which will respond to treatment if the basic management steps are corrected
- Asthma plus co-morbidities such as obesity, a breathing pattern disorder and uncontrolled allergic rhinitis
- True STRA (uncommon)

The assessment will consist of a detailed evaluation in the clinic and home and school visits, which may be virtual. The focus is on adherence, the environment (allergens and irritants) and psychosocial factors. Overall, there is a search for treatable traits [31] and, correspondingly, what the treatment should be and what treatment success will look like, in three domains. These are pulmonary (e.g., airway inflammation or infection); extrapulmonary (e.g., obesity) and social/environmental, of which this last is the domain wherein the reason for poor treatment response most often lies.

### 2.2. Can They Take the Prescribed Treatment?

Proper technique with the medication delivery device is essential and may be suboptimal despite multiple teaching sessions. In one study of directly observed therapy by videolink, it took five weeks before the technique was corrected in a group of severe asthmatics, despite multiple previous teaching sessions in the clinic [32]. This technology can certainly be used to improve inhaler technique [33]. The problem will likely be compounded if more than a single device is needed. Related is the problem of teenagers using a metered dose inhaler without a ‘babyish’ spacer.

### 2.3. Are They Taking the Prescribed Treatment? 

Low adherence is probably the most common reason for failure to respond to therapy, and there are multiple different causes. The PAPA (perceptions and practicalities) framework is very useful [34,35] (Table 4) and can help to address the problem. The approach to a child unable to cope with an immensely complex regime is very different from that to a child who is worried about side effects.

### 2.4. Case Study

A 15-year-old girl was referred after multiple asthma attacks despite high-dose ICSs and long-acting β-2 agonists. She was atopic and had a raised FeNO and blood eosinophil count. There was a 30% improvement in first-second forced expired volume after SABAs. Although she and her parents stated adherence was >90%, she had only collected enough prescriptions for <25% of maintenance therapy, and at a home visit, the medications could not be located. With directly observed therapy, her lung function and FeNO normalized, and she had no further attacks.

The gold standard to ensure adherence is directly observed therapy (DOTS) or the use of injectable medications given under direct supervision (i.e., not by an unobserved family member). Numerous methods have been described to detect non-adherence. In our experience, direct questions and questionnaires are not useful, and indeed parents may be unaware that they are not closely supervising treatment [27]. There are a number of ways of evaluating adherence, none perfect (Table 5). We use electronic monitoring of device activation for a three-month period, obviously fully informing the family about what is being measured [36]. We measure spirometry and FeNO before and after the monitoring period. The ideal would be a device that measures activation and inspiratory flow and volume, but such devices are not readily available. In one study using electronic monitoring, we found four different patterns:

- Obstructive spirometry and elevated FeNO at the start of monitoring, good adherence during monitoring, spirometry and FeNO normal at the end of monitoring. The explanation is that this is a previously poorly adherent child who took treatment while being monitored, and it worked during the monitoring period.
- Continued low adherence and poor outcomes during monitoring. The explanation is either a child with refractory difficult asthma, a child who is unpersuadable to take therapy or a child with true STRA who has become disillusioned with taking treatments which do not work.
- Good adherence but continued poor outcomes. These are the children who have true STRA requiring beyond guidelines therapy.
- Poor adherence but good outcomes. These children have previously been overtreated, so this is rational poor adherence.

For those with poor adherence and continued poor outcomes, the key question is, what happens when treatment is actually administered? This can be answered by directly observed therapy (DOT) either in the hospital or at school. If the child has STRA, DOT will not improve outcomes.

### 2.5. Are There Important Adverse Environmental Factors? Allergens

The combination of allergen sensitization, allergen exposure and respiratory viral infection is highly predictive of admission to hospital with an asthma attack [37]. Of these, only environmental allergen exposure can be modulated. Specific IgE and/or skin prick tests can identify relevant allergens, and a nurse-led home visit may also be useful. In one study, in which children sensitized to house dust mites and admitted to hospital with an asthma attack were randomized into mite-impermeable bedding cover or placebo cover groups, the mite-impermeable group had fewer emergency visits in the succeeding year [38]. It could be argued that the intervention was too narrowly focused, and if a multipronged attack on offending allergens, such as pets for example, had been included in the study, the results could have been better. A USA study addressing multiple environmental factors over a year-long period resulted in improved asthma control in the follow-up year after the program had come to an end [39]. However it is one thing to identify important allergens, and another to convince the family to address them; families are notoriously resistant to removing much-loved pet cats and dogs. The fallacious arguments include ‘the child is no worse when he is near the dog’ and ‘we removed the cat for two weeks and he was no better, so we brought the cat back’. However, low-dose allergen challenges, deliberately too low to induce bronchoconstriction, result in worsening airway inflammation and bronchial responsiveness [40], and cat allergens in particular can be detected in the environment for months after the feline has been expelled. Unless there is a food allergy documented by blind challenge, dietary manipulation has no part in the management of severe asthma.

### 2.6. Are There Important Adverse Environmental Factors? Irritants

Although worldwide, biomass fuel exposure is probably the most important indoor irritant, in a high-income setting, tobacco and vaping are significant factors. We routinely measure urine or cotinine to detect passive and indeed active smoking. There are multiple mechanisms by which smoking both active and passive worsens asthma, including inducing steroid resistance [41].

### 2.7. Are There Important Psychosocial Issues?

The role of clinical psychology is paramount in these children. Anxiety and depression are very common. We think it is pointless to try to determine whether asthma or depression came first, and which was causative; if both are present, then both should be treated on their merits by appropriate psychological interventions. In around 10% of our children, we have one or more safeguarding concerns [42,43]. One is symptom over-reporting to try to exaggerate the severity of asthma and qualify for government benefits. Discussion with schoolteachers may be illuminating.

### 2.8. Case Study

A 13-year-old boy was referred with asthma requiring daily oral corticosteroids. However, when the school was visited, the teacher told the respiratory nurse that this child allegedly needing daily oral steroids was the fastest runner in school; was captain of field hockey; was nicknamed ‘whippet’; and did not have an inhaler at school, let alone use one. Symptoms were being exaggerated so the family could claim government benefits.

Another concerning aspect is parents who will not engage, refuse to acknowledge the severity of their child’s condition and/or insist on relying on ‘complementary’ medicine.

### 2.9. The Role of Breathing Pattern Disorders and Exercise-Induced Laryngeal Obstruction (EILO)

Breathlessness, particularly on exertion, may be due to exercise-induced bronchoconstriction (EIB), but even in known asthmatics, an abnormal breathing pattern and/or EILO may be significant contributors. EILO is caused by adduction of the vocal cords, usually during inspiration, occasionally during both inspiration and expiration. Typically the patient feels unable to fill the lungs with air and points to the site of obstruction as central. Characteristically, breathlessness comes on during exercise rather than afterwards. There may be audible stridor, which may be confused with wheeze, and of course does not respond to SABA pretreatment. The definitive diagnostic test is flexible laryngoscopy during exercise. The adducted vocal cords are directly visualized, and a videoclip is shown to the patient to explain the mechanism. This is invasive and may not be tolerated by many children; simpler and often illuminating is a videoclip of the attack recorded on a smartphone [44]. Another useful test is a cardiopulmonary exercise test (CPET) which will document the breathing pattern [45]. Treatment is based first on recognizing that the problem is not asthma and will not respond to asthma medications and secondly on breathing retraining, helped by a specialist physiotherapist and sometimes a psychologist [46]. Occasionally, surgery is helpful, in which case pre-operative exercise laryngoscopy is essential. EILO is also occasionally a late complication of laryngomalacia [47]. Breathing exercises administered by a specialist physiotherapist may be very helpful.

## 3. After the Assessment: Treat and Reassess

The next step is a multi-disciplinary team meeting to put together all the data and formulate a bespoke treatment plan. The majority will be categorized as ‘difficult asthma’ who will respond to optimizing basic management, especially supporting adherence. In some children, co-morbidities will need addressing (asthma plus). Finally, a minority will be shown to have STRA and be assessed for biologicals (below).

The success or otherwise of the bespoke treatment program will be assessed three months later. Many will have improved asthma outcomes and reduction in the prescribed dose of medications [48]. However, those who are unable or unwilling to follow the program, or do not respond for any other reason, will be categorized as refractory difficult asthma (usually persistent poor adherence despite multiple efforts to support taking regular medication) and refractory asthma plus co-morbidities (usually obesity with failed weight loss, below). These children will also be assessed for biologicals. It has been argued in some guidelines that adherence to standard therapy is a prerequisite for the prescription of biologics, but we reject this view [49]. Children should not be penalized and put at risk because their family cannot support them in taking medications.

For those with STRA and either of the refractory asthmas, the next step is assessment for eligibility for biologicals. It would also be good to be able to propose biomarkers predictive of the best choice, but paediatric data are scarce. It is also clear that paediatric STRA is not one disease but many [50]. The assumption has been that STRA results from steroid-resistant, type 2 (TH2) inflammation driven by the signature cytokines interleukin (IL)-4, -5 and -13, and indeed, the TH2 pathway may be pivotal in refractory difficult asthma. However, mechanistic data have led to re-evaluation of these hypotheses. In a bronchoscopic study, although many STRA children had airway eosinophilia on bronchoalveolar lavage (BAL) and endobronchial biopsy (EBx), many were non-eosinophilic [51]. It would seem illogical to use an anti-eosinophil strategy when eosinophils are not present! Furthermore, even in those children with eosinophilia, signature TH2 cytokines were not detected in the majority, calling into question the logic of using anti-TH2 biologics. Another group [52] showed that severe asthma was neither TH1 or TH2, but severe asthma was characterized by elevations in BAL CXCL1 (growth-related oncogene, GRO), RANTES (regulated on activation, normal T cell-expressed and -secreted, CCL5), IL-12, interferon (IFN)-γ and IL-10. IL-6 was the best discriminant from the lysate of alveolar macrophages. A third group [53] proposed the helpful conceptual framework of a spectrum involving pro-inflammatory TH1, TH2 and TH17 cytokines. At one end of the spectrum was an infection-driven TH1 and TH17 group and at the opposite end was a TH2 allergy-dominated group, but with multiple other cytokines involved across the spectrum. Infection was the dominant factor (bacterial and viral) in the TH1-TH17 group. Importantly when considering anti-IL5 monoclonals (mepolizumab, benralizumab and reslizumab, the latter not licensed for use in children), BAL IL-5 increased with age, suggesting a theoretical basis, confirmed in the MUPPITS-2 study (below), that these biologicals may be less effective in younger children. However, none of these markers and models have been validated longitudinally; this is important because sputum cellularity-based phenotypes (eosinophilic, neutrophilic, mixed, paucigranulocytic) are not stable over time [54]. Furthermore, in adult studies, peripheral blood eosinophil count is used to determine choice of biologicals, with an upper limit of normal of 300 cells/μλ, but in children, this figure is well within the normal range [55]. Additionally, blood eosinophil count may be elevated in atopic conditions such as atopic dermatitis without asthma. We have a long way to go to understand how best to use these agents in children.

## 4. Options for Biologics in Children

The licensed agents, all of which have to be given by injection, are omalizumab, which binds to circulating IgE and thus preventing it from binding to the high-affinity receptor on mast cells and basophils; mepolizumab, which binds circulating IL5; dupilumab, which blocks the receptor for IL4 and IL13; benralizumab, which blocks the IL5 receptor; and Tezepelumab, which binds the epithelial alarmin thymic stromal lympopoeitin (TSLP). Tezeplumab is only licensed for use in young people aged 12 and over; for the others, it is age six years and over. However, there are limited data for all but omalizumab even in teenagers. Before the CoVID-19 pandemic, all injections were given in hospital with careful monitoring for adverse reactions. However, if the first two injections have safely been given in hospital, we now have the rest administered at home by a parent, monitored in real time by videolink by an experienced nurse [56]. We have shown this to be safe and acceptable to families. The following sections give a brief overview of the indications for these agents; interested readers are referred to a recent review [57].

### 4.1. Omalizumab 

There is the most paediatric experience with this agent, and numerous meta-analyses, systematic reviews and real-world and randomized controlled data testify to reduced asthma attacks, improved quality of life and the ability to reduce ICS and oral steroid therapy [58,59,60]. For these reasons, most would use omalizumab as the first line in eligible children. It has the added benefit of enhancing host defence against viruses, which may be a further beneficial action [61]. Dose is determined by body weight and serum IgE level, but patients with an IgE above 1300 are ineligible. Other criteria include being adherent to standard therapy (with which we do not agree; see above), aeroallergen sensitization and an arbitrary number of attacks in the previous year (in the UK, at least four, which is illogical; see below). However, adult studies have shown that those with a high IgE who are not aeroallergen-sensitized respond to omalizumab [62,63]. Even in adults, prediction of response is difficult. An initial study suggested that those with high TH2 biomarkers (blood eosinophil count, FeNO and periostin [64]—this last not useful in children, because it is released from growing bone). This seemed logical, because high blood eosinophils and FeNO are good markers of risk [65]. A small paediatric study showed that responders to omalizumab were those who had a reduction in FeNO with triamcinolone [66] as part of the multi-domain assessment of steroid responsiveness [67]. However, the recent SOMoSA study [68] failed to confirm this and suggested that five exhaled volatile compounds and five plasma lipids were better biomarkers. However, this needs to be confirmed in a second cohort. Encouragingly, omalizumab seems to work better in those with more severe asthma [69]. Our own practice, not evidence-based, is to assess response every four months and consider whether a switch to another monoclonal, or a monoclonal holiday, is indicated. Good long-term (one- and two-year) responses have been reported in children, albeit with a greater than 10% dropout rate [70,71].

### 4.2. Mepolizumab

This was the second agent licensed for use in children. There has been one randomized, double-blind, placebo-controlled trial in inner city USA children [72]. The children had to be attack-prone and have a blood eosinophil count of at least 150 cells/μλ, well below the midpoint of the normal range. Compared with adult data such as the DREAM study [73], the results were disappointing, albeit statistically significant [74]. Reasons could have included that the blood eosinophil count entry criteria were too low and that other factors such as environmental pollution were driving attacks. In the UK, mepolizumab is available for those who have failed omalizumab treatment or are ineligible for it as next line therapy.

### 4.3. Dupilumab

This agent was initially used to treat severe eczema, but it is also very valuable in severe asthma. The year-long, randomized, double-blind, placebo-controlled trial (VOYAGE) demonstrated reduction in asthma attacks, improved quality of life and improved first-second forced expired volume (FEV1) [25]. The benefit was maintained during the year-long, open-label extension (EXCURSION) [26]. The results were much better than those seen in MUPPITS-2, and it would seem logical to use dupilumab without first trialing mepolizumab, especially in children who also have eczema. There was an increase in viral infections in the dupilumab-treated children, a point to which I return below.

### 4.4. Tezepelumab

The airway epithelium, the first point of contact with infecting and polluting agents, is capable of releasing many mediators, including the alarmins IL25, IL33 and TSLP, which in turn activate other pathways, including TH2 inflammation. Tezepelumab is the first biologic that is active not just in TH2-high asthma, but also in TH2-low—excitingly, it is active irrespective of atopic status, FeNO and peripheral blood eosinophil count [75,76]. There is a need for data from younger children before it can be recommended in this age group. However, in children aged 12 and over with apparently TH2-low asthma, tezepelumab would be the first-choice biologic.

### 4.5. Benralizumab

Extraordinarily, benralizumab has been licensed for use in children on the basis of a safety and pharmacokinetic study (TATE study) in fewer than 30 children, without any efficacy data [77]. Extrapolating from adult data (always to be done with extreme caution), a real-world study showed efficacy in those who had failed treatment with other biologics [78]. However, as a cautionary note, blood eosinophils are almost completely ablated by benralizumab and are lowered to a much greater extent than by mepolizumab or reslizumab. This is associated with an increased rate of infections, including infection-driven asthma attacks [79]. Also, dupilumab was associated with more viral infections in the VOYAGE study, albeit not serious ones, supporting that eosinophils have an anti-viral effect [80]. Eosinophils are not always harmful cells and may be anti-infective as well as having other developmentally important effects [81,82,83,84,85]. On current knowledge, benralizumab should be used with extreme caution if at all in children aged 6–11 years.

### 4.6. Options for Biologics: Summary

There is a really shameful lack of data to inform our choices in children. Even in adults, there have been no head-to-head efficacy trials, and only one paediatric trial (TREAT, a comparison of omalizumab and mepolizumab) is recruiting [86]. At the moment, we are stuck with empirical, N-of-1 therapeutic trials to determine which biological (omalizumab, mepolizumab, dupilumab, benralizumab) is best for the child with TH2-driven asthma. We need to do better. We certainly need data and treatment options for TH2-low asthma in children.

## 5. Is There a Role for Bronchoscopy?

Currently we do not perform bronchoscopy before starting biologics; we rely on the standard, non-invasive markers (IgE, FeNO, blood eosinophil count). However, there is a case for bronchoscopy in children who have not responded to biologics as expected. Neutrophilic asthma is rare in our clinic and usually betokens a missed diagnosis of chronic airway infection due to underlying persistent bacterial bronchitis or bronchiectasis. Indeed, unlike in adults, airway neutrophils (intra-epithelial) are associated with better outcomes [87]. However, for the rare cases of true neutrophilic asthma, an underlying cause should be sought (especially infection, pollution and tobacco exposure). A treatment trial of azithromycin should be considered. If bronchoscopy reveals no evidence of inflammation, it is worth re-evaluating the child to ensure the issue is not over-reporting of symptoms. There is limited evidence in children of efficacy of tiotropium in children with severe asthma, and this can be trialed [88,89]. If there is no eosinophilia detected on bronchoscopy, it would seem logical to try to reduce ICS treatment at least until eosinophilia appears.

## 6. Asthma Plus: Obesity

Obesity is increasingly becoming a pandemic. In many it is a disease of poverty—obesogenic junk food is cheap and may be the only way impoverished families can feed their children. The combination of obesity and the other fellow-travelers of poverty, such as pollution, bears heavily on children.

The relationship between obesity and asthma is complex. One hypothesis is that obesity causes airway disease. One potential pathway is via altered lung mechanics. Obesity may reduce functional residual capacity, leading to airway narrowing, and lead to reduced smooth muscle stretch and thus a reduced ability to respond to bronchodilators. Other mechanisms are via obesity driven systemic inflammation and aberrant airway and lung growth (below). Another possibility is reverse causation: airway disease causes obesity, via steroid courses and reduced activity. The association could be attributable to confounding factors such as shared genetic polymorphisms, shared prenatal factors such as maternal nutrition and shared medical comorbidities such as reflux disease and obstructive sleep apnoea. Finally, poverty is a cause of obesity and brings with it other confounders, such as tobacco smoke exposure, low socioeconomic factors, indoor and outdoor pollution exposure and overcrowding. Finally, Berkson’s bias must be remembered.

Whatever the reasons, the association is a strong one. ISAAC Phase Two methodology was used to study 8–12-year-old children (n = 10,652) from 16 affluent and 8 non-affluent centres [90]. Being overweight (OR 1.14, 0.98; 1.33) or obese (OR 1.67, 1.25; 2.21) was related to wheeze and impaired spirometry (reduced FEV1/FVC, −0.90, (−1.33%; −0.47%) in the overweight and −2.46%, (3.84%; −1.07%) in the obese. However, there was no association with any other objective markers, including atopy. A systematic review of prospective cohort studies reported an increased risk of physician-diagnosed asthma in overweight children (RR = 1.35, CI 1.15–1.58), with the risk of incident asthma increasing with increasing body mass index [91]. The ALSPAC birth cohort study found self-reported or physician-diagnosed asthma in 12.3% of 4835 children aged 7–14 years [92]. They used Mendelian randomization used to eliminate confounding and reverse causation and found that higher BMI increased the risk of an asthma diagnosis in mid-childhood.

The first issue to determine is the cause of breathlessness in an obese child. A multicentre US study enrolled 200 children aged 7–17 years with physician-diagnosed asthma, of whom 40% were overweight-obese [93]. The investigators compared subject estimates and actually measured peak flow measurements over a six-week period; the obese children significantly underestimated peak flow, and there was symptom magnification. The hypothesis was that this was on the basis of deconditioning or altered lung mechanics. In a larger study from Israel, 5984 children in an Israel lung health study, of whom 5% were obese, were recruited [94]. Physician diagnosis of asthma was reported in 7.2% of obese and 3.9% of non-obese children. The obese children had increased SABA use and reported more wheeze, but bronchodilator responsiveness was significantly more common in non-obese than obese asthmatics (51% vs. 28%). In another study, around 50% of CYP who reported being breathless had neither exercise-induced asthma nor EILO [95]. If the cause is physical deconditioning, asthma medications will not work, no matter how much the dose is escalated. If in any doubt, a CPET is performed and evidence of any post-exercise bronchoconstriction sought.

The next consideration is the nature of the asthma and what treatable traits are present. Although obesity does not protect against atopic, TH2-high disease, ‘obese asthma’ may be very different from asthma in the lean child. There may be very different airway phenotypes, and great care is needed in planning treatment. Obese asthma is a huge subject [96,97,98,99], and only an outline can be given here.

Obviously the best treatment for obesity is weight loss, but all too often children struggle to achieve this. Bariatric surgery may be appropriate in extreme cases [100], after detailed assessment by a specialist team, and surgery is associated with improved outcomes in adults.

The obese airway may be characterized by dysanapsis. There are a number of definitions of dysanapsis [101], but the most common is based on spirometry. FEV1 is normal, forced vital capacity (FVC) is above normal, and the ratio FEV1/FVC is thus reduced. The anatomical explanation is that the airways are of normal calibre but greater length because the lungs are large, and although Poiseuille flow is never actually established within the airways because of the repeated branching of the airways, the Poiseuille equation shows that a longer tube has a greater resistance. Dysanapsis may be related to intra-uterine nicotine exposure [102] or rapid weight gain in the first two years of life [103], but in many cases no cause can be determined. Dysanapsis is common in obesity, but also seen in lean children and is associated with more asthma attacks and oral corticosteroid use [104].

Obesity does not of course convey protection against atopic allergic asthma, and some but not all obese asthmatics will respond to standard treatment with ICSs and LABAs. Certainly, any TH2 inflammation should be identified [105] and treated along standard lines before trialing more experimental treatments, but TH2 inflammation may not be present [106]. However, obesity is a systemic inflammatory condition, and in at least some obese asthmatics, the airway may be targeted by systemic IL-6 [107]. In these adults, IgE and eosinophil count are not related. There are anecdotal cases of successful treatment with the anti-IL6 monoclonal tocilizumab in two children with IL4Rα polymorphisms [108]. An adult study in mild asthma (a condition in which a monoclonal would never be used!) suggests that tocilizumab has no effect on allergen-induced bronchoconstriction [109]. Further trials are ongoing. There is also increasing evidence of inflammatory and airway smooth muscle cells having different properties in the obese asthmatic [110,111]. In summary, the inflammatory landscape in the obese asthmatic may be very different compared to the lean counterpart and is a fruitful subject for future work.

The interactions between the gut and airway microbiome are complex and poorly understood, but there is increasing evidence that both microbiota and their interactions are altered in obesity and asthma, alone or in combination. This another area of difference between obese and lean asthma that is poorly understood and a fruitful area for future research [112].

In summary, the obese child with difficult asthma represents a complex clinical issue, likely to become more common as the prevalence of obesity rises in the general population. Obesity is certainly associated with more admissions to hospital and intensive care unit stays [113] and is a cause of steroid resistance [114,115]. The same critical approach outlined above is needed, especially the need to be sure that breathlessness is due to asthma. As far as is possible, the airway pathology should be delineated, and it should not be assumed to be due to T2 inflammation and treatment should not be escalated uncritically.

## 7. The Attack-Prone Asthmatic Child

An asthma attack must never be treated as a trivial inconvenience which requires no more than a short course of prednisolone, but as a major red flag and a never event, like amputating the wrong leg [12]. The major risk factor for a severe asthma attack is a previous attack [116]. There should be a detailed evaluation of the child within 72 h of the attack, to prevent another one which may be fatal. An asthma attack is a sign that the management of a chronic disease has gone wrong [117,118].

The first question is, what was the severity of the attack? Unfortunately, all too often prednisolone courses are administered without any objective evidence of the seriousness or otherwise of the attack, especially if the child is looked after in a severe asthma clinic. If the oxygen saturation is 100% in a breathless and distressed child, the cause is not an acute asthma attack. Assuming the child has suffered an asthma attack, a review should take place within 48 to 72 h. This must not be a mere tick-box exercise. It is essential to determine whether ICSs are being administered or SABAs are being over-used. There should be a detailed review of the inhaler technique. Careful consideration should be given to what has triggered the attack; as above, most are triggered by viral infection with a background of T2 inflammation. Exposure of the child to environmental allergens to which they are sensitized must be minimized, as must environmental irritants such as smoking. The asthma plan should be reviewed; was it followed, and if not, why not? Does it need to be modified?

Consideration should be given to changes in medication, either the SMART regime or the use of a biological. The SMART regime (single inhaler for maintenance and relief) uses a combination of budesonide and the fast-onset, long-acting β-2 agonist formoterol, usually delivered in a Turbohaler. Planned doses may be administered once or twice a day, with rescue therapy as needed [119,120]. This strategy prevents potentially fatal overdosing on SABAs. Indeed, even in mild asthma, replacing SABAs with AIR (the same combination of inhaled medication, with the acronym meaning anti-inflammatory reliever) gives better asthma outcomes with a lower dose of ICSs that standard step 2 treatment [121]. SMART/AIR is particularly valuable for teenagers, who are notoriously non-adherent, and the asthma plan needs to be modified for SMART [122]. There is a need for more evidence in children aged less than 12 years; fortunately, such trials are ongoing [123,124]. If, despite a focused intervention after an attack, the child has a second attack, then a biological is firmly indicated, the choice depending on eligibility and local licensing.

## 8. Future Research Needs

As has been documented, there are many gaps in our knowledge which need to be addressed in future research. We need to know more about biomarkers for responses to specific biologics in individual children and probably progress from merely counting cell numbers to determining activation status [125]. Other than for omalizumab, what is lacking in children is long-term safety and efficacy data so we can weigh risks and benefits more accurately. In adults, twice-yearly injections of the anti-IL5 monoclonal depemokinab have shown promise [126] and could have a role in children, but this needs to be tested. Above all, we need a better understanding of the developmental pathology of severe asthma; without knowledge of pathology, true personalized medicine is not possible.

## 9. Conclusions: The Child with Symptoms Suggestive of Asthma Which Do Not Respond to Asthma Medications

Overwhelmingly, the right response is to ensure that the simple basic steps have been got right. Escalating treatments without consideration of why standard pre-existing treatments which should work but do not is not sensible. Instead, the wise paediatrician goes back to the beginning, the correctness or otherwise of the diagnosis of asthma, and then systematically dissects out factors which may impact treatment response, including poor adherence, adverse environmental factors and psychosocial issues. This approach will mean that many will not need biologicals but are able to be controlled on standard therapy. However, for those with STRA, and those with refractory difficult asthma who will not take standard medications, the new biologicals should be considered. We need more data if we are to personalize the choice of biologicals for the individual child. Current choices are anti-IgE (omalizumab), anti-IL5 (mepolizumab, no efficacy data for benralizumab), anti IL4/13 (dupilumab) and anti-TSLP (tezepelumab). This last is the only biological licensed for TH2-low asthma. For the appropriate children, these are life-changing. We need more paediatric data, especially biomarkers which are predictive of successful treatment.

## Figures and Tables

**Table 1:** Moving the diagnosis of asthma beyond just taking a history. There is no single test or combination of tests which is the gold standard.

Method	Test	Comment
Objective documentation of wheeze	Wheeze heard by physician Wheeze recording on mobile telephone (Futuristic) e-stethoscope on parental mobile telephone	‘Wheeze’ often used by families to describe non-specific noises
Variable airflow obstruction	Acute response to short-acting β-2 agonist if airway obstruction present (spirometry, impulse oscillometry) Home peak flow monitoring including response to exercise and short-acting β-2 agonist Exercise test Histamine or methacholine challenge	Histamine and methacholine challenge testing rarely needed; used to rule out asthma as a cause of symptoms
Atopic status	History of eczema, allergic rhinitis, food allergy Total and specific IgE to relevant allergens Skin prick testing to relevant allergens	Non-atopic asthma is well described, but in children, most severe asthma is atopic
Airway inflammation	Blood eosinophil count Exhaled nitric oxide Induced or spontaneously expectorated sputum eosinophilia Bronchoscopy, bronchoalveolar lavage and endobronchial biopsy	Blood eosinophil count and exhaled nitric oxide may be elevated in non-asthmatic, atopic children Reserved for really severe asthma in tertiary centres

**Table 2:** Pointers on the history and examination which suggest an alternative, non-asthma diagnosis.

Points in the History	Comment	Points on Examination	Comment
Very sudden onset of symptoms	Consider foreign body aspiration	Digital clubbing	Often overlooked. Consider CF, PCD, bronchiectasis and chILD
Prominent upper airway symptoms	Can be misinterpreted as wheeze	Nasal polyps	CF most common cause, also seen in PCD
Symptoms from first day of life	Consider congenital abnormality and PCD	Large tonsils, marked rhinitis	Are symptoms being misinterpreted as wheeze? Consider PCD
Chronic wet cough > 4–8 weeks duration	Not due to asthma; exclude CSLD	Signs of weight loss	Eliminate other respiratory and systemic disease depending on context
Continuous and unremitting symptoms	Even those with severe asthma usually have periods of remission	Unusually severe chest deformity	Consider CSLD
Fever and weight loss	Exclude tuberculosis	Crackles	Coarse: consider CF, PCD, bronchiectasis Fine: consider chILD
Vomiting, back arching when feeding	Consider gastro-oesophageal reflux	Stridor, fixed monophonic wheeze	Fixed large airway obstruction
Symptomatic when eating or drinking	Consider unsafe swallow	Signs of cardiac and systemic disease	Full and careful physical examination mandatory
Anything suggestive of cardiac or systemic disease	White differential diagnosis including immune deficiency		

Abbreviations: CF, cystic fibrosis; chILD, childhood interstitial lung disease; CSLD, chronic suppurative lung disease; PCD, primary ciliary dyskinesia.

**Table 3:** Criteria for the diagnosis of severe asthma in children [21,22].

Severe asthma	BDP ≥ 800 mcg/day (age 6–12 years) or ≥2000 mcg daily (age > 12) PLUS (or failed trials of) Long acting β-2 agonist Leukotriene receptor antagonist Theophylline EITHER to maintain control OR with poor control despite this therapy
Uncontrolled asthma: ≥1 of:	Poor symptom control (e.g., ACT persistently < 20) ≥2 systemic corticosteroid courses ≥ 3 days/year ≥1 serious attack requiring hospital admission, ICU stay or IPPV Persistent airflow limitation FEV_1_ Z score < 1.96 after corticosteroid trial and acute bronchodilator administration

Abbreviations: ACT, asthma control test; BDP, Beclomethasone dipropionate; FEV_1_, first second forced expired volume; ICU, intensive care unit; IPPV, intermittent positive pressure ventilation.

**Table 4:** The perceptions and practicalities (PAPA) framework in the assessment of non-adherence [34,35].

PAPA Framework	Problem	Proposed Solutions
Practicality	Forgetfulness ± chaotic lifestyle	Simplify regime Mobile phone alerts Directly observed therapy at school
Practicality	Lack of parental supervision	Identify the problem, which is often not appreciated Directly observed therapy at school
Practicality	Complex regime	Simplify; consider once-daily combination of ICSs and LABAs or SMART regime
Practicality	Visibility—not wanting to appear different	Consider regimes which do not require medication to be taken out of the home
Practicality	The family cannot afford medications which have to be purchased in high-income countries because there is no universal health coverage	Obtain charity funding or benefits Use the cheapest generic that can be found
Perception	Lack of understanding of correct usage	Asthma education
Perception	Discarding ICSs because no immediate gratification	Asthma education
Perception	Concerns about side-effects	Asthma education

Abbreviations: ICSs, inhaled corticosteroids; LABAs, long-acting β-2 agonist; SMART, single inhaler for maintenance and relief.

**Table 5:** Methods of assessing adherence.

Adherence Tool	Comment
Asking the child and family, including questionnaires	Non-judgemental questions, assuming that non-adherence is normal may help, but this is generally unreliable; questionnaires seem to work better in a research context.
Prescription records	Picking up a prescription does not mean it has been cashed or, if cashed, has been correctly utilised. If the prescription has not been utilised, non-adherence is certain
Home visit (face-to-face or virtual)	If the medication cannot be located or is out of date or is stockpiled in original boxes, poor adherence is likely
Monitoring of medication blood levels	Applicable for theophylline or prednisolone, but neither often used for maintenance therapy; blood levels of inhaled medications not routinely available
Electronic activation monitor, e.g., smart inhaler	Can only say if and when the device has been activated, not whether used correctly
Electronic devices with inspiratory flow sensor	Not yet widely available and usually only measures peak inspiratory flow, not inspired volume. Probably the gold standard
Directly observed therapy—a response means previous non-adherence	At school, but only if child attends regularly. Does not work if child does not go to school; not on weekend or during school holidays Using mobile phone: very resource intensive Admission to hospital: very resource intensive
Response to a single dose of intramuscular triamcinolone	Tests if the child is steroid-sensitive; good response highly suggestive of previous poor adherence
